# Protective effect of Polygonatum sibiricum Polysaccharide on D-galactose-induced aging rats model

**DOI:** 10.1038/s41598-020-59055-7

**Published:** 2020-02-10

**Authors:** Shaoyan Zheng

**Affiliations:** Pharmacy Department, Foshan Women And Children Hospital Affiliated to Southern Medical University, Foshan, P.R. China

**Keywords:** Experimental evolution, Molecular medicine

## Abstract

The polysaccharide of Polygonatum sibiricum (PSP)is one of the main active ingredients of Polygonatum Polygonatum in Liliaceae. It has anti-tumor, anti-aging, immune regulation, and anti-oxidative effects. Recent studies have shown that the Klotho gene and fibroblast growth factor-23 (FGF-23) have a common receptor, which is closely related to aging and highly expressed in kidney and meninges. Our study aimed to investigate the anti-aging effect of PSP on D-galactose-induced rats and its mechanism. D-galactose (120 mg Kg^-1^) and PSP (100 mg Kg^-1^) was used to intervene in rats, respectively. Then The changes of indexes of the natural aging-like model rats before and after PSP intervention were observed. We found that PSP could significantly improve the learning and memory abilities of rats and reverse the pathological changes of kidney tissues in rats. At the same time, PSP up-regulated the expression of Klotho mRNA and Klotho protein in the renal cortex, down-regulated the expression of FOXO3a mRNA and p-FOXO3a protein in renal tissue, and inhibited the expression of FGF-23 protein in the femur. Our studies suggest that PSP may play a role by regulating the Klotho-FGF23 endocrine axis, alleviating oxidative stress, and balancing calcium and phosphorus metabolism.

## Introduction

Aging should be an irreversible, comprehensive, and gradual loss of the function of each cell and organ tissue in the body. It is the body in the disease and function of the high-risk state of decline. The affecting factor of aging is not merely one, but rather a result of a combination of factors synergies. Modern medical studies have shown that the essence of aging refers to the irreversible process of gradual degeneration of structure and function after biological development and maturation under normal conditions, with the increase of age, the decline of self-function, the decline of internal environmental stability and stress ability, and the gradual degeneration of structure and function. With the development of modern genetic technology, the mechanism of aging has been studied in depth. It has become one of the research hotspots in recent years to search for active substances with an anti-aging effect in Nature by using the mechanism of aging.

Klotho gene, which is an anti-aging gene, was first discovered by Kuro-o^1^ in 1997, and then Xiao^2^, Kurosu^3^, Yamamoto^4^ and others studied the gene and its expression. The results showed that the Klotho gene and fibroblast growth factor-23 (fibroblast growth factor-23) have common receptors, which are strictly related to aging and highly expressed in kidney and meninges.

In January 2018, Chinese and American research teams published their research results in Nature. The crystal structure of the ternary complex of anti-aging protein alpha klotho-fibroblast growth factor receptor 1c (fibroblast growth factor receptor 1c) - fibroblast growth factor 23 (fibroblast growth factor 23) was analyzed, and the mechanism of its regulation of aging was elucidated^5^. The above studies and reports indicate that the Klotho-FGF23 signaling pathway is closely related to aging.

In China, Polygonatum sibiricum is traditional Chinese medicine. Modern pharmacological research has found that Polygonatum sibiricum has the effects of anti-aging^6^, hypoglycemic, hypolipidemic, anti-inflammatory^7^, etc. Polygonatum sibiricum polysaccharides (PSP) is one of the main bioactive components of Polygonatum sibiricum. It has been reported that PSP contains five components, i.e., PSW1B - b, PSW2A- 1, PSW3A - 1, PSW4A, and PSW5B. High-performance liquid chromatography analysis of PSP indicates that it is composed of Rhamnose, Arabinose, Xylose, Mannose, Glucose, and Galactose. Galactan and Mannan are the primary components, with alpha (1, 4) glycosidic bond or 1–6 glycosidic bond connected^8^. The relative molecular mass of PSP is about 7457, and the dispersion coefficient is 1025^9^. The results of Zhao Hongxia^10^, YI Yuxin^11^, Li Youyuan^12^, and others show that Polygonatum can play an anti-aging role by eliminating SOD, improving learning and memory ability, improving the ultrastructure of Cal region in the hippocampus or increasing the activity of granules. However, how Polygonatum Polysaccharide exerts its anti-aging effect, and whether it is related to the Klotho-FGF23 signaling pathway has not been reported.

D-galactose is currently recognized as an aging reagent, which can be used to replicate the animal aging model^13,14^. It can simulate the normal aging process, which can not only lead to memory and cognitive impairment but also change the similar significant aging characteristics, such as MDA content, SOD activity, and GSH-Px activity. So D-galactose is widely used in anti-aging and tissue and organ damage research. In this way, the primary purpose of this article is to explore the anti-aging effect of Polygonatum polysaccharide and its possible mechanism by establishing the D-galactose aging model.

## Results

### PSP can improve the spatial memory ability of rats induced by D-galactose

**Figure 1:**
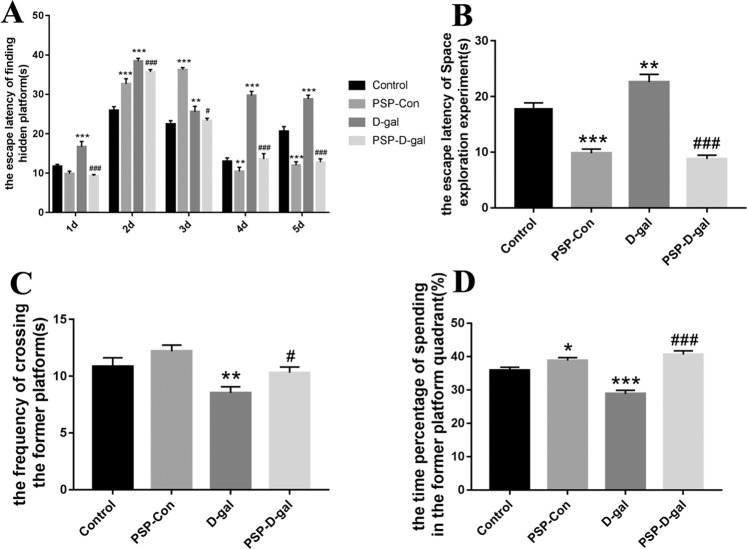
PSP improves the spatial memory ability of rats induced by D-galactose. (**A**) Effects of PSP on the escape latency in the Hidden Platform Search Experiment of D-galactose-induced rats and PSP shortens latency time (p < 0.001). (**B–D**) Effect of PSP on space exploration test of D-galactose-induced rats. (**B**) PSP reduces the escape latency of the Space exploration experiment (p < 0.001). (**C**) PSP enhances the frequency of crossing the former platform (p < 0.05). (**D**) PSP increases the time percentage of spending in the former platform quadrant (p < 0.05). ^*^p < 0.05, **p < 0.01, ^***^p < 0.001 vs. Control group. ^#^p < 0.05, ^##^p < 0.01, ^###^p < 0.001 vs. D-gal group.

The results showed that the escape latency of the D-gal group was significantly longer than that of the Control group, that of PSP-Con group was shorter than that of the Control group, and that of PSP-D-gal group was shorter than that of D-galactose group (Fig. 1A). In space exploration experiment, the escape latency of D-gal group was longer than that of Control group (Fig. 1B), while the number of times of crossing the original platform and the percentage of residence time in the original platform quadrant were lower than that of Control group (Fig. 1C,D). the escape latency of the PSP-D-gal group was shorter than that of the D-gal group; the number of times crossing the original plateau quadrant and the percentage of residence time in the original plateau quadrant was higher than that of the D-gal group (Fig. 1B,D).

### PSP can improve learning and memory ability of rats induced by D-galactose

**Figure 2:**
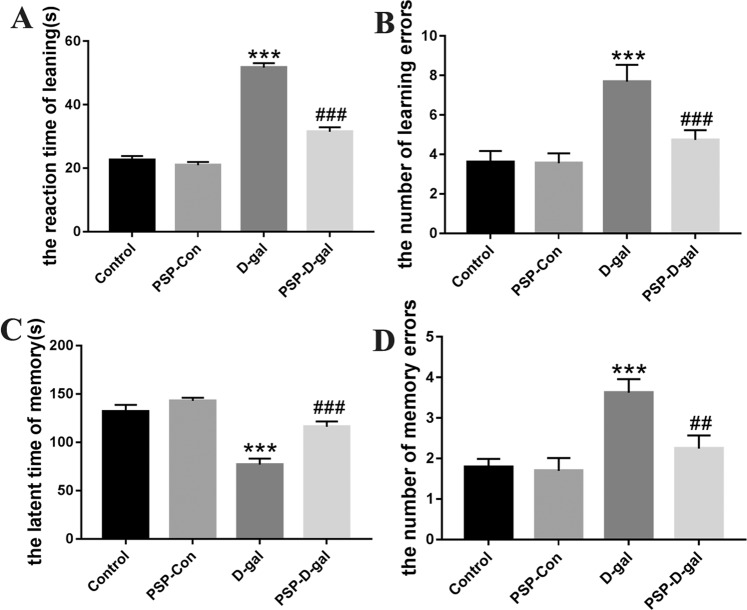
Effects of PSP on the performance of rats in the step-down test. (**A**) PSP reduces the reaction time of leaning (p < 0.001). (**B**) PSP decreases the number of learning errors (p < 0.01). (**C**) PSP prolongs the latent time of memory (p < 0.001). (**D**) PSP reduces the number of memory errors (p < 0.01). *p < 0.05, **p < 0.01, ***p < 0.001 vs. Control group. ^#^p < 0.05, ^##^p < 0.01, ^###^p < 0.001 vs. D-gal group.

In step-down experiment, the learning response time of rats in the Control group, PSP-D-gal group and PSP-Con group was significantly shorter than that in the D-gal group (Fig. 2A), and the latency time of memory was significantly prolonged (Fig. 2C), and the number of learning and memory errors was significantly reduced (Fig. 2B,D).

### PSP can increase thymus index and spleen index of rats induced by D-galactose

**Figure 3:**
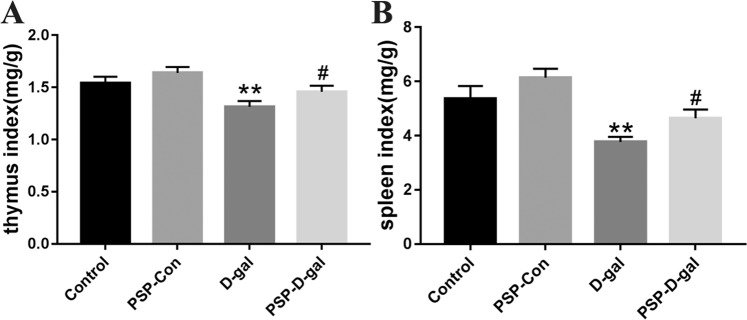
PSP increases the thymus index and spleen index of rats induced by D-galactose. (**A**) PSP increases thymus index (p < 0.05). (**B**) PSP improves spleen index (p < 0.05). *p < 0.05, **p < 0.01, ***p < 0.001 vs. Control group. ^#^p < 0.05, ^##^p < 0.01, ^###^p < 0.001 vs. D-gal group.

The results showed that the thymus index and spleen index of D-gal group were significantly lower than those of Control group, while the thymus index and spleen index of PSP-Con group were higher; compared with D-gal group, the thymus index and spleen index of PSP-D-gal group were significantly higher than those of D-gal group (Fig. 3A,B).

### PSP can improve renal function of rats induced by D-galactose

**Figure 4:**
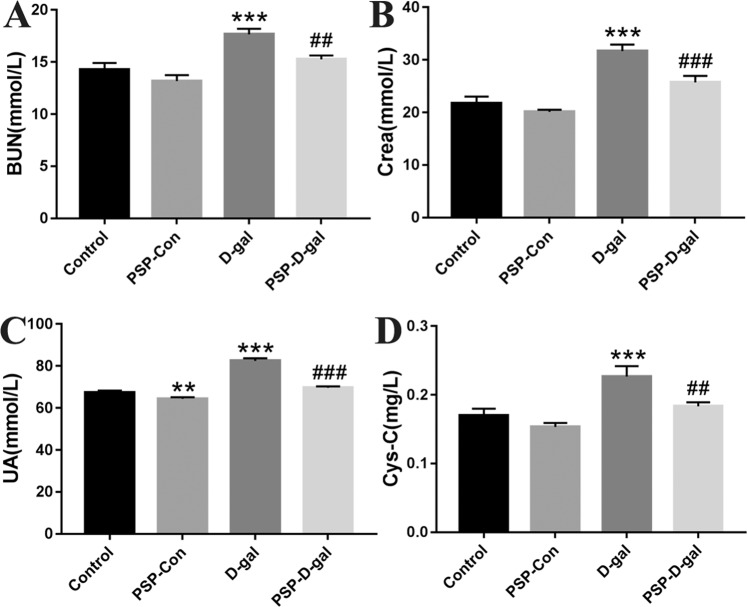
PSP improves renal function in rats induced by D-galactose. (**A–D**) PSP reduces the contents of BUN, Crea, UA and Cys-C, respectively. *p < 0.05, **p < 0.01, ***p < 0.001 vs. Control group. ^#^p < 0.05, ^##^p < 0.01, ^###^p < 0.001 vs. D-gal group.

Compared with the Control group, the renal function indicators BUN, Crea, UA and Cys-C in the D-gal group increased significantly; the renal function indicators in the PSP-Con group decreased; compared with the D-gal group, the renal function indicators BUN, Crea, UA and Cys-C in the PSP-D-gal group decreased significantly (Fig. 4A–D).

### PSP can improve calcium and phosphorus metabolism in rats induced by D-galactose

**Figure 5:**
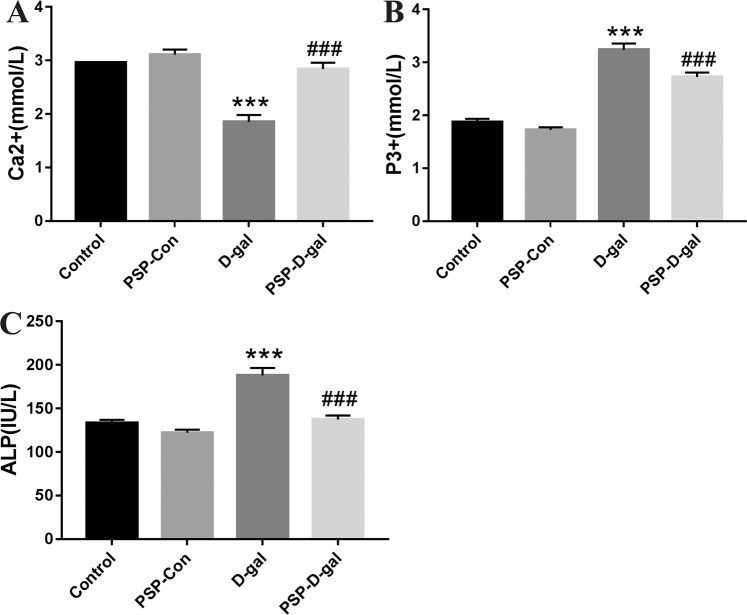
PSP improves calcium and phosphorus metabolism in rats induced by D-galactose. (**A**) PSP increased the content of Ca2+ in the blood of senile rats (p < 0.001). (**B–C**)PSP decreased the content of ALP and P3 + in the blood of senile rats (p < 0.001), respectively. *p < 0.05, **p < 0.01, ***p < 0.001 vs. Control group. ^#^p < 0.05, ^##^p < 0.01, ^###^p < 0.001 vs. D-gal group.

Compared with the Control group, ALP, P3+ increased and Ca2+ decreased in the D-gal group; Ca2+ increased and ALP, P3+ decreased in the normal PSP group; compared with the D-gal group, ALP, P3+ decreased and Ca2+ increased in the PSP-D-gal group (Fig. 5A–C).

### PSP can reduce NO and NOS in blood of rats induced by D-galactose

**Figure 6:**
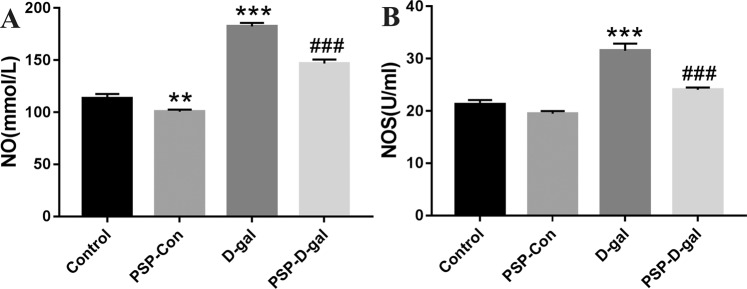
Effects of PSP on NO and NOS in blood of rats. (**A**) PSP decreases NO content in blood of rats (p < 0.001). (**B**) PSP reduces NOS content in blood of rats (p < 0.001). *p < 0.05, **p < 0.01, ***p < 0.001 vs. Control group. ^#^p < 0.05, ^##^p < 0.01, ^###^p < 0.001 vs. D-gal group.

Compared with the Control group, the D-gal group can significantly increase NO and NOS in blood, while the PSP Control group can reduce them (Fig. 6A,B); compared with the D-gal group, the PSP-D-gal group can significantly reduce NO and NOS in blood (Fig. 6A,B).

### PSP can improve the ability of antioxidant damage of kidney tissue in rats induced by D-galactose

**Figure 7:**
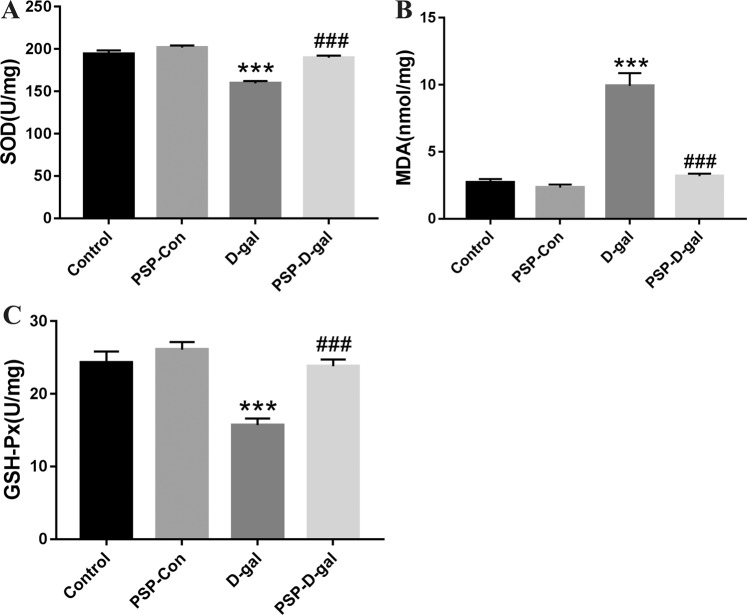
Effects of PSP on SOD, MDA and GSH-Px in kidney tissues of senile rats induced by D-galactose. (**A**) PSP increases SOD content in kidney tissue of rats (p < 0.001). (**B**) PSP enhances GSH-Px activity in kidney tissue of rats (p < 0.001). (**C**) PSP decreases MDA content in kidney tissue of rats (p < 0.001). *p < 0.05, **p < 0.01, ***p < 0.001 vs. Control group. ^#^p < 0.05, ^##^p < 0.01, ^###^p < 0.001 vs. D-gal group.

Compared with the Control group, the SOD content and GSH-Px activity in kidney homogenate of D-galactose model rats were significantly decreased, while the MDA content was significantly increased. Compared with D-gal group, the SOD content and GSH-Px activity of PSP-D-gal group increased significantly, but the MDA content decreased significantly (Fig. 7A–C).

### PSP can inhibit renal histopathological changes in rats induced by D-galactose

**Figure 8:**
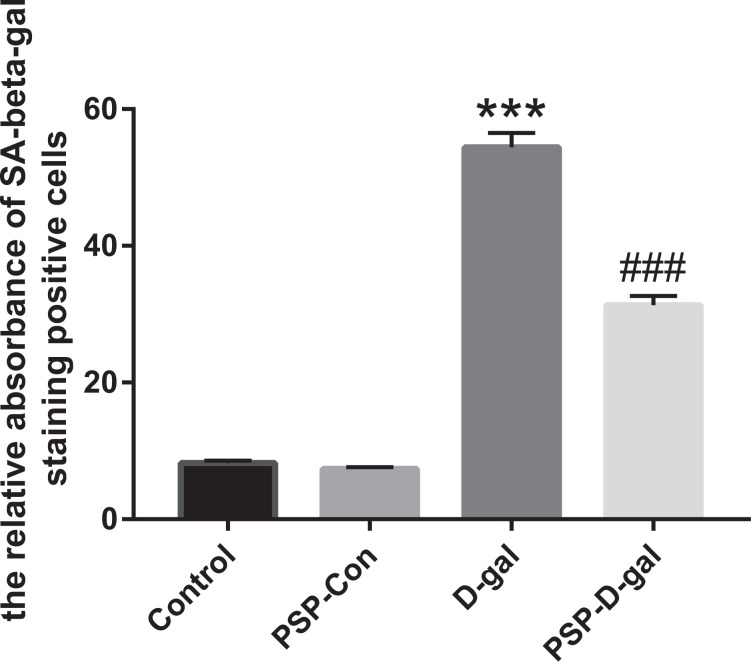
Effect of PSP on the dyeing of beta-galactosidase in kidney tissue of rats in D-gal group. PSP decreases the relative absorbance of SA-beta-gal positive cells and the volume of positive particles (p < 0.001). *p < 0.05, **p < 0.01, ***p < 0.001 vs. Control group. ^#^p < 0.05, ^##^p < 0.01, ^###^p < 0.001 vs. D-gal group^.^.

**Figure 9:**
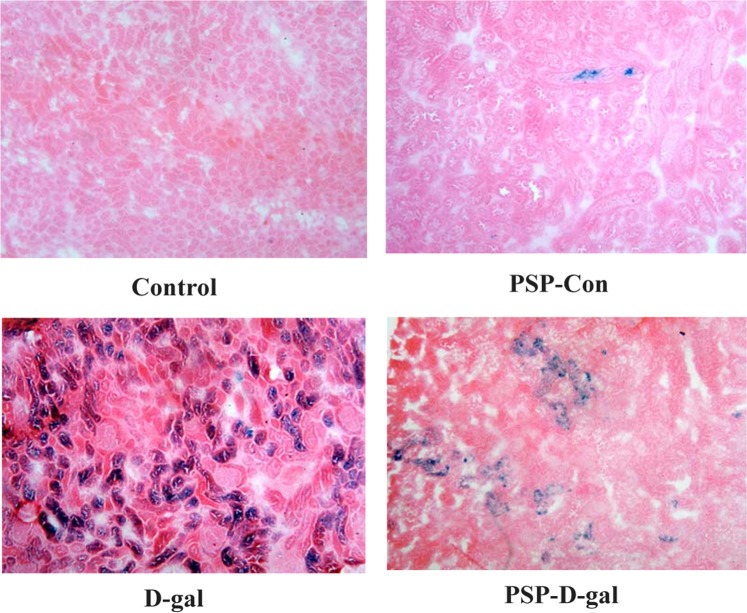
Effect of Polygonatum Polysaccharide on aging of kidney cells in D-galactose model rats (SA-beta-gal staining, ×100). In control group and PSP-Con group, the relative absorbance of SA-beta-gal positive cells in renal tubules was low; in D-gal group, the relative absorbance of SA-beta-gal positive cells was significantly higher; in PSP group, the relative absorbance of SA-beta-gal positive cells was significantly lower.

The positive granules of beta-galactosidase staining were blue. The positive cells of SA-beta-gal staining only appeared in the renal tubules and rarely expressed in the glomeruli. The relative absorbance of SA-beta-gal staining positive cells in normal renal tubules was low; the relative absorbance of SA-beta-gal staining positive cells in D-gal group was significantly increased, and the positive particles were large; the relative absorbance of SA-beta-gal staining positive cells in PSP-D-gal group was significantly decreased, and the particle size was small (Figs. 8 and 9).

### PSP can increase the expression of Klotho mRNA and decrease the expression of Akt1 and FOXO3a mRNA

**Figure 10:**
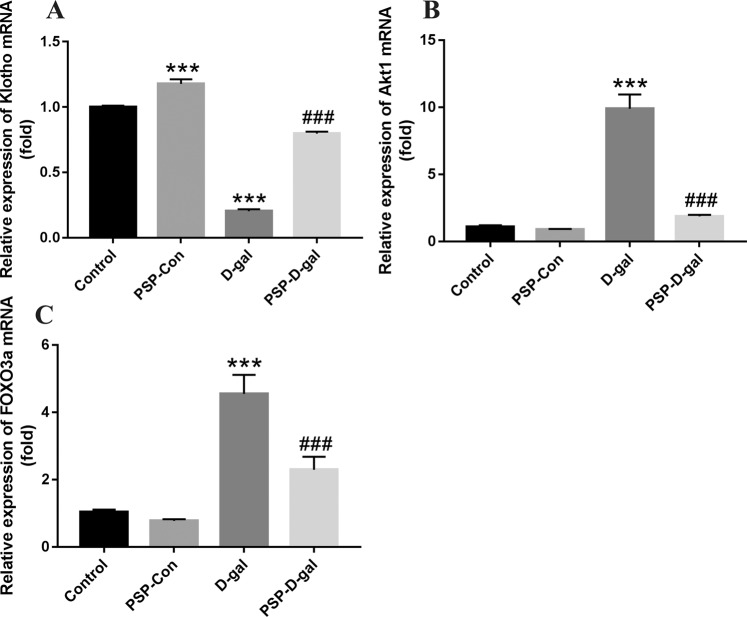
Effects of PSP on the expression of Klotho, Akt1 and FOXO3a mRNA in rats. (**A**) PSP increases the expression of Klotho mRNA (p < 0.001). (**B**) PSP decreases the expression of Akt1 mRNA (p < 0.001). (**C**) PSP reduces the expression of FOXO3a mRNA (p < 0.001). *p < 0.05, **p < 0.01, ***p < 0.001 vs. Control group. ^#^p < 0.05, ^##^p < 0.01, ^###^p < 0.001 vs. D-gal group.

Compared with the Control group, Klotho mRNA in kidney tissue of D-gal group were significantly decreased, while Akt1 and FOXO3a were significantly increased. Compared with D-gal group, Klotho mRNA of PSP-D-gal group were significantly increased, and Akt1 and FOXO3a were significantly decreased (Fig. 10A–C).

### PSP can increase the expression of Klotho, Akt and FOXO3a protein, and decrease the expression of FGF23, p-Akt and p-FOXO3a protein

**Figure 11:**
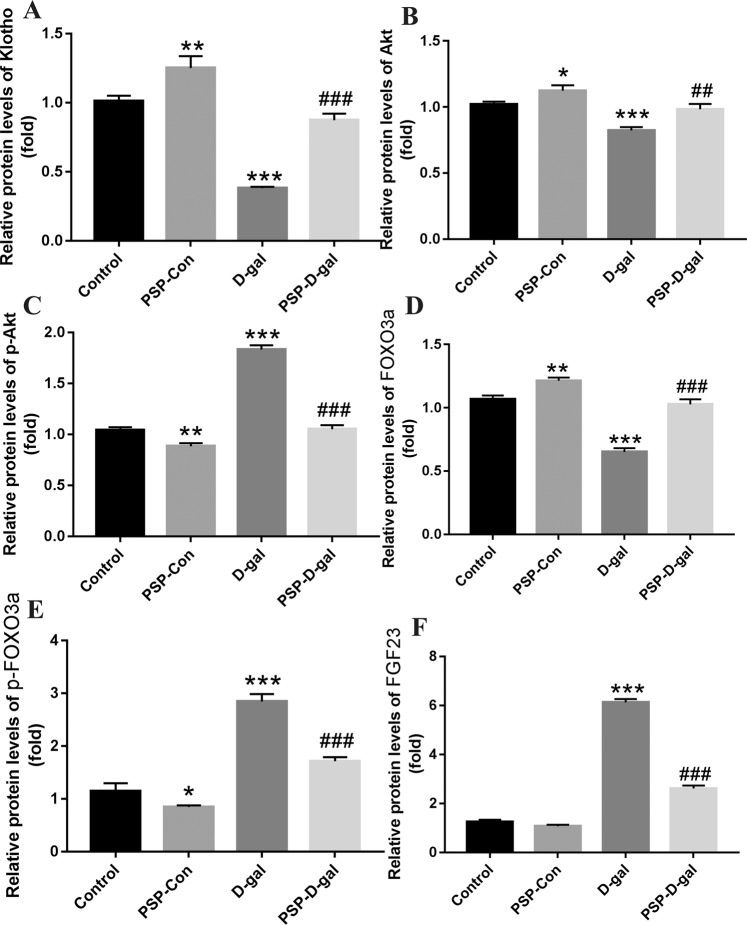
Effects of PSP on the expression of Klotho, Akt, FOXO3a, FGF23, p-Akt and p-FOXO3a proteins. (**A**) PSP increases the expression of Klotho protein (p < 0.001). (**B**) PSP enhances the expression of Akt protein (p < 0.01). (**C**) PSP decreases the expression of p-Akt protein (p < 0.001). (**D**) PSP increases the expression of FOXO3a protein (p < 0.001). (**E**) PSPdecreases the expression of FGF23 protein (p < 0.001). *p < 0.05, **p < 0.01, ***p < 0.001 vs. Control group. ^#^p < 0.05, ^##^p < 0.01, ^###^p < 0.001 vs. D-gal group.

**Figure 12:**
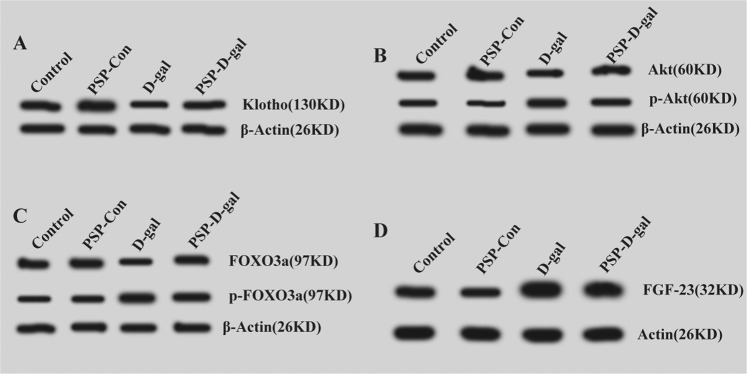
Western blot was used to detect the expression of Klotho, Akt, p-Akt, FOXO3a, p-FOXO3a and FGF23 protein.

Compared with the Control group, the expression of Klotho, Akt and FOXO3a in kidney was significantly decreased, the expression of p-Akt and p-FOXO3a was significantly increased, and the expression of fibroblast growth factor 23 in femur was significantly increased in D-gal group. Compared with D-gal group, the expression of Klotho, Akt and FOXO3a in kidney was significantly increased, the expression of p-Akt and p-FOXO3a was significantly decreased, and the expression of fibroblast growth factor 23 in femur was significantly decreased in PSP-D-gal group. (Figs. 11A–F and 12)

## Discussion

Aging is a process in which all kinds of functions of the body decrease, homeostasis function decreases, and the body gradually loses its ability to adapt to the environment with the increase of age. Recent studies on the causes and mechanisms of aging have shown that the continuous oxygen free radical reaction in cells and tissues may be the main cause of aging^15^. D-gal is currently recognized as a deteriorating agent of oxidative damage, which can induce degenerative changes similar to natural aging, such as biochemical changes similar to natural aging, low immune function, abnormal gene expression and regulation, decline of cell growth and reproductive ability, degenerative changes of brain tissue, decline of learning and memory, etc. Animal aging model prepared by D-gal is similar to natural aging^16–19^.

In this study, D-galactose was used to establish the rat model. The latency of the model rats to find the water maze platform was longer than that of the Control group (P < 0.05), suggesting that there were obstacles in learning and memory reproduction and discrimination. The latency of the PSP-D-gal group and PSP-Con group was shorter than that of the Control group (P < 0.05), which indicated that the ability of learning and memory reproduction and discrimination of senile rats after the early intervention of PSP was better than that of the D-gal group. At the same time, in the step-down experiment, the learning response time of rats in the Control group, PSP-D-gal group, and PSP-Con group was significantly shorter than that in the D-gal group, and the latent memory time was significantly prolonged. The number of learning and memory errors significantly reduced. It also showed that PSP could significantly enhance the learning and memory abilities of rats. Besides, as a vital immune organ, the spleen plays a crucial role in the healthy development of the immune function. PSP can increase the spleen index, which indicates that PSP can enhance the immunity of the body. Aging-related beta-galactosidase staining is a well-recognized biological marker and reliable evidence for cell aging^20,21^. The results showed that the relative absorbance of senile beta-galactosidase-positive cells in renal tissue increased significantly compared with the Control group, indicating that the senile cells had senescence. Compared with the D-gal group, the relative absorbance of senile beta-galactosidase-positive cells in the PSP-D-gal group decreased significantly, suggesting that PSP has the effect of delaying cell senescence.

GSH-PX and SOD are important oxygen free radical removal enzymes *in vivo*, and their activities can indirectly reflect the ROS level in cells. MDA is produced by the interaction of oxygen free radicals and lipids and has cytotoxicity. It can cross-link proteins and nucleic acids, thus inactivating proteins, causing cell dysfunction and reflecting the damage degree of ROS to organisms. By studying galactokinase-deficient GM00334 fibroblasts, Elzi DJ *et al*.^22^ have revealed that excessive D-galactose metabolism produces a large number of ROS in cells, which results in oxidative stress damage and induces cell senescence, which proves that oxidative stress damage can cause cell senescence.

In addition, As a kind of highly active free radical, nitric oxide (NO) has the characteristics of oxidation-reduction. It is easy to interact with oxygen-free radicals to produce more active free radical peroxynitrite (ONOO-) *in vivo*, resulting in cytotoxicity and damage^23,24^, leading to aging. Because no is very unstable *in vivo* and its half-life is very short, it is difficult to detect it directly, so in the study, the level of NO is indirectly reflected by detecting the activity of nitric oxide synthase (NOS). Nitric oxide synthetase (NOS) is an essential enzyme for no synthesis. There are three main types *in vivo*^25,26^: the neuron type (nNOS) mainly exists in the central nervous system; the endothelial type (eNOS) mainly distributes in the endothelial cells; the inducible nitric oxide synthase (iNOS) mainly exists in the mammalian nucleated cells^27^. nNOS and eNOS need the participation of calcium/calmodulin to catalyze *in vivo*. After activation, the enzyme activity lasts for a short time, and the amount of no synthesized and released is tiny. However, iNOS does not rely on calcium/calmodulin to catalyze *in vivo* and has a long half-life. Once iNOS is expressed, a large amount of NO can be produced continuously. Therefore, it can be considered that iNOS is the primary basis for excessive no production *in vivo*^28^. At the same time, in recent years, many scholars have found that iNOS is related to the aging of internal organs in animal experiments. Eva Siles^29^ and other scholars found that the level of iNOS in the cerebellum of rats increased significantly with age, and the level of tyrosine nitrating protein and the activity of NOS also increased accordingly. It is believed that the increase of iNOS expression makes the synthesis of NO increase the transformation of peroxynitrite ONOO-, thus increasing the nitration of protein and affecting the aging of the cerebellum. Therefore, we think that the detection of iNOS can better reflect the synthesis of NO. Thus, in this study, total NOS was determined by the determination of iNOS. Both of them can combine with oxygen free radicals to cause aging.

In this study, GSH-PX, SOD activity, MDA, NO, NOS content were used as indicators of changes in the oxidative stress level of the Control group, PSP-Con group, D-gal group, and PSP-D-gal group. The results showed that D-gal could decrease the antioxidant capacity and increase the ROS content of cells, while PSP could resist the aging-induced by D-gal, increase the activity of GSH-PX and SOD, and reduce the contents of MDA, NOS and NO.

At present, the changes of NO with age are not consistent in different literature reports. Some studies suggest that the content of NO decreases with age^30–32^. But at the same time, a large number of studies have confirmed that^33–36^, after aging, there will be a significant increase in the content of NO and the activity of NOS. The results of our study showed that the content of NO increased with the increase of age, that is, the content of NO in renal tissue of D-gal group was significantly higher than that of the Control group, PSP could reduce the content of NO and the activity of NOS in renal tissue of rats, suggesting that PSP could produce anti-aging effect by inhibiting the increase of NO and NOS, and its mechanism might be related to blocking the combination of NO and NOS with oxygen-free radicals.

It has also proved that the model of D-gal group is effective, and related to oxidative stress injury and ROS toxicity. Because of its good antioxidant effect, PSP can improve the oxidative stress state of cells and enhance the antioxidant capacity of cells, to achieve the effect of delaying aging.

Fibroblast growth factor 23 (fibroblast growth factor 23) is a cytokine involved in blood phosphorus metabolism. It was first discovered and named by Yamashita^37^ in 2000. Its N-terminal contains a skin for secretion. Therefore, this secretory function in the blood is not autocrine or paracrine. It is an atypical member of the fibroblast growth factor family^38^. Fibroblast growth factor-23 gene excision can lead to a wide range of premature aging-like manifestations in humans, including growth retardation, hunchback, muscle wasting, infertility, atherosclerosis, extensive soft tissue calcification, multiple organ system atrophy, biologic disorders of calcium and phosphorus metabolism, emphysema, osteoporosis and severe life expectancy^39^.

Klotho protein is an aging-related factor, which can enhance the affinity of fibroblast growth factor 23 with fibroblast growth factor Rlc. The coordinated action of Klotho and fibroblast growth factor Rlc constitutes fibroblast growth factor 23 receptor^40^. Recent studies have shown that fibroblast growth factor receptor (fibroblast growth factor receptor, fibroblast growth factor receptor)/a-Klotho complex regulates blood phosphorus metabolism^5^. This study showed that PSP could decrease the content of ALP and P3+ and increase the content of Ca2+ in the blood of rats, suggesting that the anti-aging effect of PSP might be related to the regulation of calcium and phosphorus metabolism.

In addition, the existing studies have shown that Klotho protein can negatively regulate phosphatidylinositol 3-kinase/Akt signaling pathway, thereby increasing FOXO3-mediated manganese superoxide dismutase expression and enhancing the body to play an antioxidant stress role^41^. And recent studies also found that the high expression of Klotho protein can inhibit the process of downstream Akt acting on serine residue 473 (Ser473) and threonine residue 308 (Thr308) in the substrate and phosphorylating them^42^. In addition, Klotho protein, as a necessary auxiliary factor for FGF23 to mediate receptor activation, can activate the downstream signal pathway, mediate its biological activity^43^, negatively regulate downstream Akt activity and phosphorylation expression of its substrate FOXO3a, to play a role in delaying aging^44^. Our results showed that the D-gal group’s relative protein expression of Klotho, Akt and FOXO3a in kidney was significantly decreased, the expression of p-Akt and p-FOXO3a was significantly increased, compared with the Control group, which was consistent with the conclusion of previous studies. In addition, the relative expression level of Klotho mRNA was lower and Akt1 and FOXO3a mRNA was higher in the D-gal group than that in the Control group (P < 0.05). Compared with the D-gal group, the relative expression of Klotho mRNA in the PSP-Con group and PSP-D-gal group increased, while that in Akt1 and FOXO3a decreased (P < 0.05). Our results suggest that the early intervention of PSP can up-regulate the expression of Klotho mRNA in the aging tissues of D- gal group rats, and down-regulate the expression of Akt1 and FOXO3a mRNA. At the same time, the results also showed that PSP could decrease the content of ALP, P^3 +^, and increase the content of Ca^2 +^ in the blood of rats, suggesting that the anti-aging effect of PSP may be related to the regulation of calcium and phosphorus metabolism.

At the same time, Western blot results also showed that compared with the Control group, the expression of Klotho, Akt, and FOXO3a protein in kidney and femur of D-gal model group was significantly lower, and the expression of FGF23, p-Akt, and p-FOXO3a protein significantly increased; the expression of Klotho protein in kidney tissue of D-gal group was decreased, the phosphorylation inhibition of downstream Akt protein was weakened, and the expression of p-Akt protein was increased, which led to the downstream FOXO3a gene translation. The expression of p-FOXO3a protein mainly increased. Most of the p-FOXO3a protein entered the cytoplasm and lost its activity. The expression of total FOXO3a protein in the nucleus was still decreased, which resulted in the relatively weak antioxidant capacity of the body and the aging symptoms of cognitive decline in rats.

Compared with the D-gal group, the Klotho, Akt, and FOXO3a protein expression in kidney and femur significantly increased, while the expressions of FGF23, p-Akt, and p-FOXO3a protein significantly decreased, which was consistent with the changes of gene expression. After treatment with PSP, the expression of Klotho and FOXO3a in kidney tissues of the PSP-Con group and PSP-D-gal group increased, while the expression of Akt and FOXO3a downstream decreased. The expression of Klotho protein in kidney tissue of PSP-Con group and PSP-D-gal group increased, the phosphorylation inhibition of downstream Akt and FOXO3a protein strengthened, and the total FOXO3a protein expression was still higher than that of D-gal group. Although the expression of FOXO3a decreased in the PSP-D-gal group, the expression of p-FOXO3a was mainly decreased, and the expression of total FOXO3a was significantly increased compared with the D-gal group. In addition, the expression of fibroblast growth factor-23 decreased with the increase of Klotho protein expression in different groups, while the expression of fibroblast growth factor-23 increased with the decrease of Klotho protein expression. This indicates that there is an interaction between Klotho and fibroblast growth factor-23, which jointly regulates the anti-oxidative stress of the body, improves the cognitive impairment of rats, and plays a role in protecting the kidney function, thus delaying aging. However, Due to the limitation of the project, the pharmacokinetic study on the anti-aging effect of PSP was not carried out. Still, a large number of materials on polysaccharide and PSP were especially consulted. The existing studies showed that the acidic polysaccharide and glycoprotein in the polysaccharide had immunoactivity^8^. For this study, the PSP was mainly composed of psw1a-1 and psw3a-1, which belonged to the acidic polysaccharide, psw1b-b belongs to neutral galactose, psw4a and psw5b belong to the glycoprotein. Therefore, we speculate that psw1a-1 and psw3a-1 belong to the acidic polysaccharide, and psw4a and psw5b belong to the glycoprotein. It is still necessary to further study the pharmacokinetics and anti-aging components of PSP.

In conclusion, PSP plays an important role in delaying aging by acting on the Klotho-FGF23 endocrine axis, increasing blood calcium, reducing blood phosphorus and alkaline phosphatase, up-regulating the expression of Klotho mRNA and K1otho protein in renal cortex and inhibiting the level of fibroblast growth factor-23 protein in femur, so as to alleviate oxidative stress and improve the renal function of aging model rats induced by D-galactose.

## Materials and Methods

All experimental methods were performed in accordance with the relevant guidelines and regulations.

### Animals and treatment

Male Sprague–Dawley (SD) rats weighing between 28 and 32 g were ordered from Shanghai Experimental Animal Center, which were housed in a 12–12-hour light-dark cycle environment. The room temperature was controlled between 20 and 22 °C, and animals were allowed to access food and water freely. All experimental procedures were conducted in conformity with institutional guidelines approved by the Chinese Association of Laboratory Animal Care. All experimental protocols were approved by the Institutional Animal Care and Use Committee in Southern Medical University, Guangzhou, China.

Before the experiment, rats were divided into 4 groups (n = 30 for each group) using computer generated random numbers: the Control group, D-gal group, PSP-D-gal group, and PSP-Con group. Among them, D-gal group: referring to the relevant literature, rats were subcutaneously injected with D-galactose (120 mg·Kg^-1^) for 42 days. PSP D-galactose group: PSP (100 mg·Kg^-1^) was injected intraperitoneally for 35 days from the eighth day of D-galactose injection. PSP-Con group: rats were subcutaneously injected with normal saline on the eighth day and intraperitoneally injected with PSP (100 mg Kg^-1^) for 35 days. Control group: rats were subcutaneously and intraperitoneally injected with normal saline for 42 days. Every index was measured the next day after the modeling and administration were completed.

### Reagents and instruments

Polygonatum Polysaccharide (98% purity, Shaanxi Ciyuan Biotechnology Co., Ltd.), D-galactose (Beijing Solebo Technology Co., Ltd.), saline solution (Anhui Shuanghe Pharmaceutical Co., Ltd.), SOD kit, MDA kit, NO test kit, NO synthase test kit, ATP content test kit, Glutathione Peroxidase (GSH-PX) Kit, which were purchased from Nanjing Jiangcheng Bioengineering Institute. Reagents for RNA extraction include DNA/RNA sample protectant, RNA-free water, universal RNA extraction kit, PrimeScriptTM RT Master Mix (Perfect Real Time) RR036A for reverse transcription, SYBR Premix Ex Taq TM II RR820A, all of which were purchased from TaKaRa Company. RIPA cell lysate (Beijing Solebo Biotechnology Co., Ltd.); protease inhibitor benzyl sulfonyl fluoride (PMSF; Beijing Solebo Biotechnology Co., Ltd.); phosphatase inhibitor (Beijing Solebo Biotechnology Co., Ltd.); quinoline formic acid protein quantitative kit (Biyuntian Biotechnology Co., Ltd.); hypersensitive electrochemiluminescent reagent (Biyuntian Biology Technology Co., Ltd.). The first anti-Akt, phosphorylated Akt (p-Akt), FOXO3a, phosphorylated FOXO3a (p-FOXO3a), Beta-Actin (β-Actin) and the second anti-Akt were purchased from Santa Cruz Biotechnology Company, USA. Klotho was purchased from Abcam Company, USA. Digital display constant temperature magnetic stirrer (Jiangsu Jintan Kanghua Electronic Instrument Manufacturing Plant); Frozen desktop centrifuge (Germany EPPENDORF Company); High-speed homogenizer (Shanghai Specimen Model Factory).

## Method

### Morris water maze experiment

The spatial memory ability of rats in each group was tested by using the Morris water maze test, which was performed on the 2nd day after model replication or drug injection.

#### Hidden platform search experiments

Rats were placed into the wall of the pool in the same random order from four entry points every day for 120 seconds. No platform was found in the prescribed time, and the escape period was 120 seconds. Rats were guided to the platform. The residence time of each rat platform was 30 seconds. The average escape latency of rats in each group was measured four times a day for five days.

#### Spatial search experiment

On the 6th day, the platform was withdrawn, and two quadrants far from the platform were selected to enter the water. The time of rats in the target quadrant and the times of crossing the target within 120 seconds were measured.

### Mouse platform jumping test

After the last administration, the rats were placed in a platform jumper. Ten minutes later, the rats were electrified (36 V). When the rats were shocked, they jumped onto the platform and recorded the number of shocks and the reaction time within 5 minutes. After 24 hours of adaptation to the environment, the rats were placed on the jumping platform again until they were knocked off the platform and recorded as latent time questions. The number of electric shocks within 3 minutes was recorded as memory scores.

### Thymus and spleen index

After the last administration, the rats were weighed. After 30 minutes, the eyeballs were taken for blood, dislocated, and executed. The thymus and spleen were taken for weighing, and the indexes of thymus and spleen were calculated. Calculating formula: thymus index = thymus mass (mg)/mouse body mass (g), spleen index = spleen mass (mg)/mouse body mass (g).

### Detection of renal function

The serum was prepared from the eyeball blood of rats in each group. Renal function indexes such as blood urea nitrogen (BUN), muscle intoxication (Crea), uric acid (UA), and cystatin C (Cys-C) were detected by the automatic biochemical instrument.

### Detection of serum indicators in rats

After 30 minutes of last administration, extracting the eyeball blood for 1 ml and then keeping for 30 minutes, the concentration of NO, NOS, Ca2+, P3+, ALP in serum was determined by centrifugation for 10 minutes. The supernatant was taken as serum. The contents of NO, NOS, Ca2+, P3+, ALP in serum were determined according to the instructions of the colorimetric kit.

### Detection of oxidative and antioxidant capacity of kidney tissue

The kidney homogenate of 10% of rats was prepared, and the supernatant was collected after centrifugation for 10 minutes. BCA protein concentration assay kit was used to detect the protein concentration in the supernatant of each group. The related indicators were detected according to SOD, MDA, and GSH-Px kit instructions.

### RT-PCR Detection of Klotho, Akt1, FOXO3a mRNA expression level

The hippocampus and kidney tissues of rats in each group were separately taken. After grinding with liquid nitrogen to a fine powder, the Buffer RL pyrolysis solution containing 50 DTT solution was added to the hippocampus and kidney tissues, and then centrifuged for 3 minutes at 4 C for 12 000 r/min.

Total RNA in hippocampus and kidney was extracted strictly according to the kit instructions. The concentration and purity of total RNA were determined by nucleic acid protein analyzer. According to the concentration of total RNA, the DNA was retranscribed and used as a template for PCR amplification.

Akt1, FOXO3a, Klotho specific primers were designed and synthesized by Bao Bioengineering (Dalian) Co., Ltd. β-Actin was purchased from Bao Bioengineering (Dalian) Co., Ltd.

The primer sequence is as follows:(1) the forward primer of Aktl: CATCGTGTGGCAG-GATGTGTA, the reverse primer: ACCTGGTGTCAGTCTCAGAGGTG, the amplified fragment length is 80pb; (2) the forward primer of FOXO3a: TGCTAAGCAGGC-CTCATCTCAA, the reverse primer: AACCTCTAAACCCATCACTCTC-CAC, the amplified fragment length is 159pb; (3) the forward primer of Klotho: ATCTTTGAC-CAGACCTTGGAATGAA, the reverse primer: GAACATCATGGCATCCAAG-CAC, the amplified fragment length is 125 pb. The reaction conditions were as follows: pre-denaturation at 95 for 30 seconds, pre-denaturation at 95 for 5 seconds, and post-denaturation at 60 for 30 seconds. A total of 40 cycles were used for PCR. The expression levels of Akt1, FOXO3a and Klotho were expressed by 2^−△△Ct^ method.

### Detection of Klotho protein and fibroblast growth factor 23 protein in kidney and femur by Western blot

The kidney and femur of rats were taken out from the refrigerator at −80 °C, and lysed with RIPA cell lysate. Protease inhibitors and phosphatase inhibitors were added to avoid protein degradation. The protein content was determined by the quantitative kit of quinoline formic acid protein after lysis. The remaining liquid was transferred to the centrifuge tube and boiled in water at 100 °C for 5 minutes. After that, it is stored in a refrigerator at −20 °C according to the device of 50 ml per tube.

The protein was separated by 10% polyacrylamide gel electrophoresis which the electrophoretic condition is 60 V for 2.5 hours. Transferred to PVDF membrane for electrophoresis at 300 ma for 1.5 h and then blocked for 2 hours by blocking buffer. Adding rabbit anti mouse antibody p-IRS-1, Klotho, p-FOXO3a,p-Akt, β-Actin which was diluted by primary antibody dilution buffer to the protein respectively after being rinsed three times (15 mins per time) by TBST (tris buffered saline with tween-20), shook 4 °C overnight.

The next day was rinsed 3 times (15 minutes per time) by TBST solution, added Goat anti-Rabbit Antibody II which was diluted with Secondary Antibody Dilution Buffer, and Incubated 1 H with shaking bed at 37 °C. After TBST liquid rinsed 3 times (15 minutes per time), then added Ultrasensitive electrochemiluminescent reagent solution, placed in gel imager through Image Lab software for gray-scale scan development. After development, the strips were rinsed with pure water for 10 minutes, with Removal solution of primary and secondary antibodies (strongly alkaline) for 10 minutes, rinsed with pure water for 10 minutes and then sealed with sealing solution for 2 hours. TBST solution was rinsed three times (15 minutes per time) and then diluted with anti-dilution solution. Rabbit anti-rat FOXO3a, Akt anti-first antibody were added to shake the bed for overnight. The next day, goat anti-rabbit antibody was washed three times (15 minutes per time) with TBST solution and diluted with anti-rabbit diluent, incubated in shaking bed at 37 °C for 1 hour, and then rinsed three times (15 minutes per time) with TBST solution. The total protein was scanned and developed through image Lab software by adding Ultrasensitive electrochemiluminescent reagent solution into the developer. The image was analyzed by image analysis software IPP6.0. The β-Actin was used as internal reference.

### Ethical approval

All applicable international, national, and/or institutional guidelines for the care and use of animals were followed.
